# Metabolism-associated molecular classification of gastric adenocarcinoma

**DOI:** 10.3389/fonc.2022.1024985

**Published:** 2022-11-16

**Authors:** Yuqing Ye, Wenyun Yang, Xinjia Ruan, Li Xu, Wenxuan Cheng, Mengmeng Zhao, Xin Wang, Xinyi Chen, Daren Cai, Guanjie Li, Yuhang Wang, Fangrong Yan, Xiaofan Lu, Liyun Jiang

**Affiliations:** ^1^ State Key Laboratory of Natural Medicines, Research Center of Biostatistics and Computational Pharmacy, China Pharmaceutical University, Nanjing, China; ^2^ Department of Cancer and Functional Genomics, Institute of Genetics and Molecular and Cellular Biology, CNRS/INSERM/UNISTRA, Illkirch, France

**Keywords:** gastric cancer, metabolism signatures, molecular classification, immune cell infiltration, prognostic model, immunotherapy

## Abstract

Most gastric cancers (GC) are adenocarcinomas, whereas GC is a highly heterogeneous disease due to its molecular heterogeneity. However, traditional morphology-based classification systems, including the WHO classification and Lauren’s classification, have limited utility in guiding clinical treatment. We performed nonnegative matrix factorization (NMF) clustering based on 2752 metabolism-associated genes. We characterized each of the subclasses from multiple angles, including subclass-associated metabolism signatures, immune cell infiltration, clinic10al characteristics, drug sensitivity, and pathway enrichment. As a result, four subtypes were identified: immune suppressed, metabolic, mesenchymal/immune exhausted and hypermutated. The subtypes exhibited significant prognostic differences, which suggests that the metabolism-related classification has clinical significance. Metabolic and hypermutated subtypes have better overall survival, and the hypermutated subtype is likely to be sensitive to anti-PD-1 immunotherapy. In addition, our work showed a strong connection with previously established classifications, especially Lei’s subtype, to which we provided an interpretation based on the immune cell infiltration perspective, deepening the understanding of GC heterogeneity. Finally, a 120-gene classifier was generated to determine the GC classification, and a 10-gene prognostic model was developed for survival time prediction.

## Introduction

Gastric cancer is a significant cancer worldwide, accounting for over one million new cases and an expected 769,000 deaths in 2020, ranking fifth for cancer incidence and third for the most prevalent cause of cancer-related death (1, 2). Cancer is considered to be a metabolic disease and to be a result of metabolic dysfunction. During carcinogenesis, metabolism is changed to benefit cancer cells. The best-known example of this type of metabolism is the Warburg effect, which has been observed in numerous cancer cells and tumors (3). This impact involves high glucose absorption, accelerated glycolysis, and the conversion of pyruvate to lactic acid rather than oxidative phosphorylation (OXPHOS) to produce energy under aerobic conditions (4). In addition to providing extra energy, the breakdown of glucose through glycolysis creates glycolytic intermediates for cell growth and the synthesis of macromolecules (5). It has been demonstrated that GC cells and normal cells exhibit metabolic differences not only in glucose metabolism but also in the metabolism of lipids and amino acids, which further involves changes in the key enzymes in glycolysis, mitochondrial proteins, noncoding RNAs, and proteins that regulate these factors (6). Studies have reported that metabolism-related mechanisms are involved in gastric cancer. For instance, Lin et al. (7) discovered that metabolic stress is one of the mechanisms that elevates MACC1 expression in GC, and MACC1 upregulation compensatively ensures GC growth against metabolic stress by facilitating the Warburg effect. Gao et al. (8) reported that the aminoacyl-tRNA biosynthesis pathway is upregulated in gastric cancer and that both threonyl-tRNA synthetase and phenylalanyl-tRNA synthetase play key roles in the progression of gastric cancer. In pan-cancer research (9), transcriptional metabolic dysregulation has been reported in gastric cancer.

Furthermore, metabolic reprogramming causes alterations in the tumor microenvironment that not only affect tumor cells but also influence infiltrated immune cells. For example, Poznanski et al. (10) discovered that the malfunction of human NK cells in the microenvironment of tumors is related to the inhibition of glucose metabolism by lipid peroxidation-associated oxidative stress. *In vivo*, activation of the NRF2 antioxidant pathway restored the metabolism and function of NK cells, resulting in enhanced antitumor activity. Enhanced tumor killing in the tumor microenvironment was observed in expanded NK cells reprogrammed with intact metabolic flexibility and metabolic fitness, in response to nutrient deprivation (10). Cholesterol in the tumor microenvironment increases CD8+ T cell expression of immune checkpoints and exhaustion, according to Ma et al. (11) Cholesterol-rich tumor tissues and the cholesterol content of tumor-infiltrating CD8+ T cells were positively related with increased T cell expression of PD-1, 2B4, TIM-3, and LAG-3. Immune checkpoint blockers (ICBs) have been introduced to the treatment of gastric cancer, and the FDA approved two PD-1 ICBs for treating metastatic gastric cancer. The demand for efficient biomarkers for ICBs has emerged; however, current GC classification has limited clinical utility in guiding patient treatment.

The classification of gastric cancer has been widely studied. Traditionally, the WHO classification divides tumors into papillary, tubular, mucinous, and poorly cohesive types (12). Lauren classification categorizes tumors into intestinal, diffuse, and mixed (13). Because these classification systems are morphology-based classification systems, they have not incorporated the advances in the molecular and genetic aspects of the diseases and do not provide guidance for treatment. The Cancer Genome Atlas (TCGA) subtypes were reported in 2014 (14), proposing a molecular classification dividing GC into four subtypes: tumors positive for Epstein–Barr virus (EBV), microsatellite unstable tumors (MSI), genomically stable tumors (GS), and tumors with chromosomal instability (CIN). Other studies include but are not limited to Tan et al.’s research (15) based on the genomic expression of GC cell lines, which identified two subgroups: genomic intestinal and genomic diffuse. Lei et al. (16) reported another molecular classification that classifies GC into three independent subtypes: proliferative, metabolic, and mesenchymal. In comparison to morphology-based classification systems, molecular-based systems are a large leap forward in explaining genetic heterogeneity. However, little research work has been done to develop a classification system from a metabolism perspective, and the landscape of the metabolism signatures in GC has yet to be comprehensively elucidated.

Therefore, we performed an unsupervised classification analysis based on previously reported metabolism-relevant genes. GC patients were mainly classified into four subtypes: immune suppressed, metabolic, mesenchymal/immune exhausted and hypermutated. Based on analyses from various angles, including the evaluation of the prognostic value, correlations with metabolic signatures, immune infiltration, clinical characteristics, and drug sensitivity of the GC subclasses, we depicted the characteristics of each GC subtype and compared them with previous classifications. Finally, a 120-gene classifier was generated to determine the GC classification, a 10-gene prognostic model was developed for the survival time of the patients, and the risk score groups displayed distinctive prognostics. The subtypes exhibited substantial prognostic differences, which suggests that the metabolism-related classification has clinical significance. We hope this approach from a metabolism perspective adds new information to the understanding of gastric cancer.

## Materials and methods

### Data and sample collection

Transcriptome HTSeq count data from patients diagnosed with gastric adenocarcinoma were retrieved from Genomic Data Commons (GDC). Data from the TCGA-STAD project were downloaded using the ‘TCGAbiolinks’ R package (17). Primary malignancies were chosen from fresh-frozen samples. GENCODE27 was used to translate Ensembl IDs for mRNAs to gene symbols. Patient survival data were downloaded from the PanCanAtlas project and filtered for STAD tumor type. A total of 348 TCGA patients had their expression data matched with survival data. The number of fragments per kilobase million (FPKM) was converted into transcripts per kilobase million (TPM) values, which were comparable to microarray expression values.

Additionally, the microarray data of Gene Expression Omnibus (GEO) cohorts GSE15459, GSE84426 and GSE84433 and their clinicopathological data were retrieved. Eight patients’ expression data in GSE15459 were excluded due to missing survival data. As a result, there were 192, 76 and 357 patient expression profiles matched with survival data for GSE15459, GSE84426 and GSE84433, respectively. The TCGA dataset, GSE15459 and GSE84426 were combined as the training set for robustness, and GSE84433 was used as the validation set.

The pathological stage status of GSE84433 and GSE84426 was derived based on the T stage and N stage information according to AJAA 7th edition, making it consistent with TCGA and GSE15459 for further analysis. The algorithm ‘combat’ (18) was chosen to eliminate the possibility of batch effects from nonbiological technological biases between various datasets, and the ‘sva’ R package was used (19) Principal component analysis was performed to examine the batch effect before and after correction.

### Identification of GC subclasses

First, a previously published list of 2,752 metabolism-relevant genes encoding human metabolic enzymes and transporters was obtained for downstream analysis (20). Next, metabolism-relevant genes with a median absolute deviation (MAD) less than 0.2 were excluded, and univariate Cox regression assessing the associations of all metabolism-relevant genes with overall survival (OS) was conducted using the R package ‘survival’. Eventually, 460 candidate genes were obtained with significant prognostic value (P < 0.05).

Subsequently, unsupervised NMF clustering methods were performed using the ‘NMF’ R package (21) on the training set and the validation set using the same candidate genes. The optimal value of k was determined at the point where the magnitude of the cophenetic correlation coefficient began to fall (22). Subclass mapping (SubMap) analysis was then used to determine whether the subclasses identified in the two above datasets were correlated (23). A t-distributed stochastic neighbor embedding (t-SNE)-based approach was then used to validate the subtype assignments using the mRNA expression data of the above metabolic genes.

### Gene set variation analysis

Gene set variation analysis (GSVA) is a nonparametric unsupervised gene set enrichment method that can estimate the score of a certain pathway or signature based on transcriptomic data (24). The 113 metabolism-relevant gene signatures were obtained from a previously published study (9), and by using the ‘GSVA’ R package, each sample received 113 scores corresponding to metabolism signatures. Subsequently, differential analysis was conducted based on the 113 metabolism scores using the ‘limma’ package (25) in R software, and the pathways with an absolute log2-fold change > 0.2 and adjusted P < 0.05 were defined as differentially expressed pathways. Then, the mutually exclusive pathways for each subtype were defined as the signature pathway. The gene signatures of the 11 oncogenic pathways were retrieved from a publication (26), and the enrichment score for each subtype was calculated.

### Estimation of immune infiltration

Microenvironment cell population counter (MCPcounter) was used to evaluate the abundance of eight immune cell populations and two stromal cell populations (27). In addition, single-sample GSEA (ssGSEA) (28) was used to estimate the enrichment of T cell subtypes and fibroblast subtypes. Furthermore, immune scores and stromal scores were calculated by applying the ESTIMATE algorithm (29), which can reflect the abundance of stromal and immune cells.

### Enrichment analysis of DEGs

The differentially expressed genes (DEGs) among GC subclasses were identified using the ‘limma’ package. Genes with an absolute FC > 1.5 and adjusted P < 0.05 were defined as DEGs. The gene set of ‘h.all.v7.5.1.entrez’, ‘c5.go.bp.v7.5.1.entrez’ and ‘c2.cp.kegg.v6.2.entrez.gmt’ were downloaded from the Molecular Signatures Database (https://www.gsea-msigdb.org) and were employed for the functional and pathway enrichment analysis using the ‘clusterProfiler’ R package (30), and the significance threshold was set at an adjusted P < 0.05.

### Somatic mutation and copy number variation analysis

Gene mutation data of TCGA-STAD patient samples were downloaded from cBioPortal (https://www.cbioportal.org/). The R package ‘maftools’ (31) was used to analyze the association between subtypes and tumor mutation. The copy number variation data of TCGA-STAD tumor samples were downloaded from Firehose (http://gdac.broadinstitute.org/). The Genomic Identification of Significant Targets in Cancer (GISTIC2.0) algorithm was utilized to classify the copy number variant genes (32), and the default settings were used.

### Prediction of the response to immunotherapy

Tumor Immune Dysfunction and Exclusion analysis (TIDE) was introduced to predict patient response to immunotherapy (33, 34). The default cutoff value of zero was used for categorizing whether a response was to be expected. The data from gastric cancer patients treated with immunotherapies (35) were used to predict the immunotherapy efficacy of our subclasses by measuring the similarity of gene expression profiles between our subclasses and gastric cancer patients based on SubMap analysis (Gene Pattern) (23).

### Construction of the prognostic model

The DEGs were first subjected to a feature selection process with the R package ‘Boruta’ (36). Three hundred and sixty-two confirmed genes were then combined with the 460 previously defined prognosis-relevant metabolism genes, and the lasso-Cox model was fitted. The final model has ten genes. The risk score was calculated as the summation of the product of the coefficients and the expression value of the genes. The median risk score was used to divide the patient population into high- and low-risk groups.

### Statistical analyses

All statistical analyses were carried out by R 4.1.0. The Kruskal–Wallis test was used to compare more than two groups. The Wilcoxon test was used to compare two groups. The correlation between two continuous variables was measured using Pearson’s correlation coefficient. Kaplan–Meier curves were generated for the survival data, and the log-rank test was used to detect the difference. For all statistical analyses, a two-sided P value less than 0.05 was considered statistically significant.

## Results

### NMF identifies four subclasses in GC

**Figure 1:**
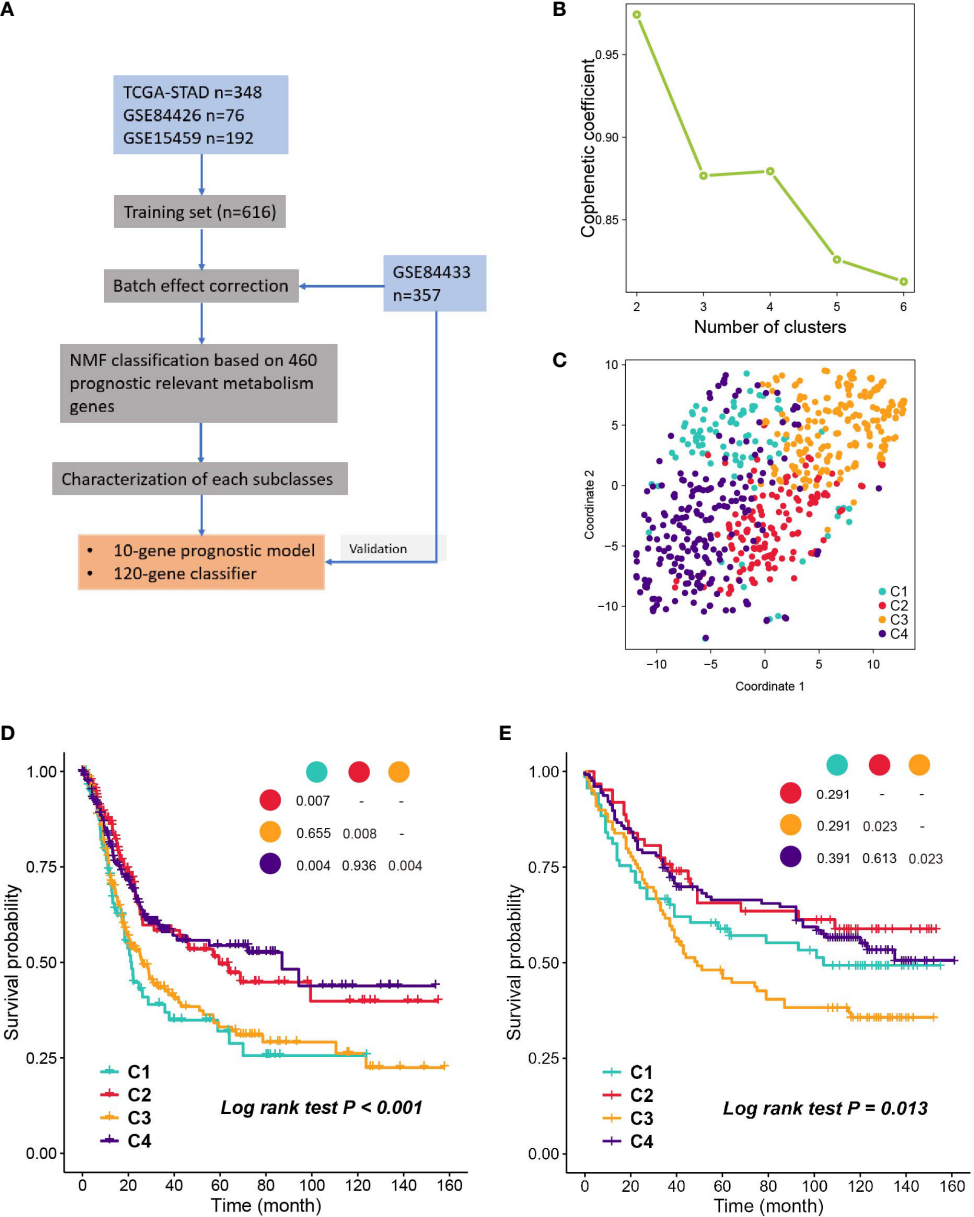
Identification of subclasses with NMF. **(A)** The flow chart of the study. **(B)** Cophenetic coefficient of NMF for k= 2 to 6, k=4 is chosen as the optimal. **(C)** Visualization of the clustering result in the training set with t-SNE. **(D, E)** Kaplan- Meier plot of OS of subclasses in training set and validation set.

A flow chart was developed to systematically describe our study (**Figure 1A**), and the clinical characteristics of patients from different cohorts are listed in **Table S1**. NMF was performed on the 460 prognostic related metabolism genes (**Table S2**). Cophenetic coefficients were calculated to determine the optimal number of clusters. When k = 4, the cophenetic coefficient drops (**Figure 1B**), and the consensus matrix heatmap maintain distinct boundaries, suggesting stable and robust clustering for the samples (**Figure S1B**). In parallel, we performed another independent clustering analysis on 357 GC samples from the GSE84433 dataset. SubMap analysis was then conducted to determine whether the clusters identified in the two independent analyses were correlated, and the results showed that C1-C4 subclasses in the training set were highly correlated with the corresponding counterpart in GSE84433 (**Figure S1D**), suggesting that there were four distinct metabolic gene expression patterns in GC. To visualize the subclasses’ assignments, we performed t-SNE to decrease the dimension of features (**Figures 1C**, **S1C**), and the four-subtype designation was well clustered. Hence, k = 4 was eventually chosen. Survival analysis was performed on the subclasses, and a significant difference was observed in both datasets (log-rank test P < 0.001, **Figure 1D**, training set; P = 0.013, **Figure 1E**, testing set). Subtypes C2 and C4 showed better overall survival than C1 and C3.

### Correlation of the GC subclasses with metabolism-associated signatures

**Figure 2:**
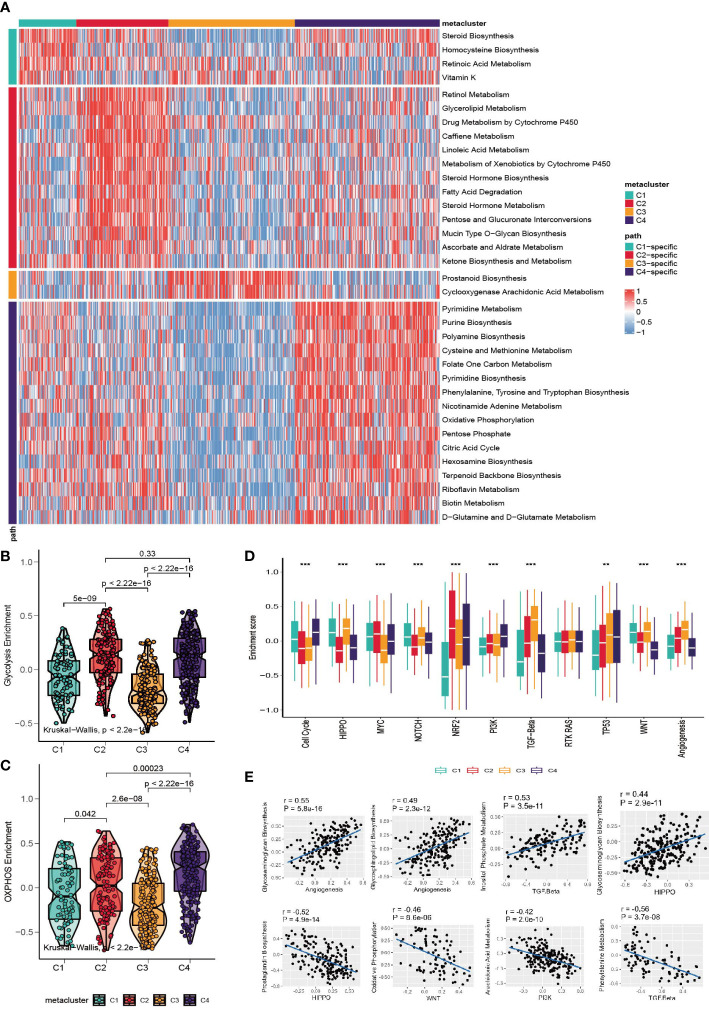
Association between metabolism signature and GC subclasses. **(A)** Heatmap of subclass-specific metabolism signatures. **(B, C)** Comparison of glycolysis and OXPHOS level among C1-C4. **(D)** Enrichment of oncogenic pathway signatures among C1-C4 (*p < 0.05; **p < 0.01; ***p < 0.001). **(E)** Pearson correlation between oncogenic pathways and metabolism pathway.

Considering that the classification was based on metabolism-relevant genes, we further investigated the metabolic characteristics for each subclass. First, 113 metabolic processes were quantified using the GSVA R package. Then, differential analysis was conducted to identify subclass-specific metabolism signatures. The results revealed that C2 and C4 were more metabolically active because they had 13 and 16 metabolism signatures, respectively, whereas C1 and C3 had only 4 and 2 signatures (**Figure 2A**). Glycolysis and OXPHOS did not appear to be metabolic signatures, suggesting that they were overexpressed in more than one subclass. Since they are the most well-studied metabolic pathways and have a substantial impact on glucose metabolism and the TME, we explored the status of glycolysis and OXPHOS among the subclasses. C3 had the lowest glycolysis and OXPHOS levels, whereas C4 had higher levels in these pathways (**Figures 2B, C**).

To further investigate the characteristics of subclasses, the enrichment of eleven oncogenic pathways was quantified using GSVA (**Figure 2D**). C1 was highly enriched in HIPPO, WNT, NOTCH and the cell cycle; C2 was highly expressed in MYC and NRF2; C3 had high values of TGF-beta and angiogenesis; and C4 had high levels of the cell cycle, TP53 and PI3K. The landscape between the metabolism and oncogenesis pathways was visualized with a heatmap (**Figure S2A**). The highest and lowest correlated oncogenesis and metabolism pathways are shown in **Figure 2E**.

### Correlation between GC subtypes and tumor-infiltrating immune cells

**Figure 3:**
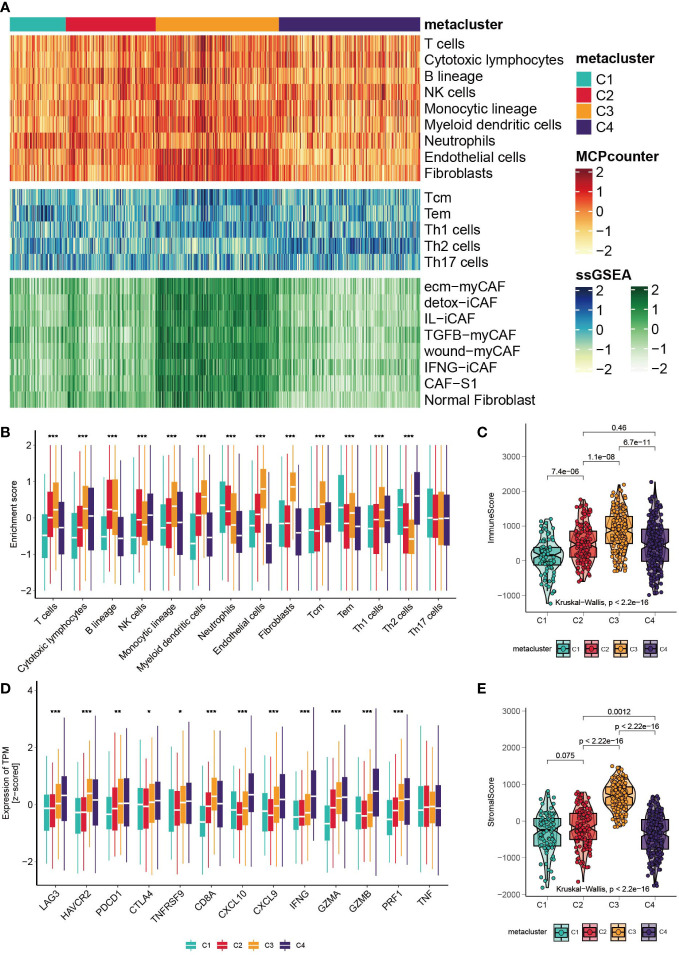
Correlation between GC subtypes and tumor infiltrated immune cells. **(A)** Heatmap of tumor infiltrated immune cells, T cell subtypes and fibroblast subtypes. **(B)** Boxplot of the enrichment level of infiltrated immune cells (*p < 0.05; **p < 0.01; ***p < 0.001). **(C)** Boxplot of the expression level of immune related genes. **(D, E)** Boxplot of the stromal score and immune score.

The landscape of tumor-infiltrating immune cells is displayed in **Figure 3A**, and a quantitative comparison of the immune cells was performed (**Figure 3B**). Except for neutrophils and effector memory T cells (Tems), C1 has poor expression of immune cells, indicating neutrophil-induced immune suppression (37). Subtype C3 is characterized by a high abundance of T helper type 1 (Th1), central memory T (Tcm), myeloid dendritic cells, cytotoxic lymphocytes and B cells. Notably, the expression of endothelial cells and fibroblasts was highly elevated in C3, indicating enhanced endothelial-mesenchymal transition (EndMT) (38). Based on a previously published fibroblast subtype signature, we further investigated which of the subtypes contributed to the high expression of fibroblasts in C3. Impressively, all fibroblast subtypes were highly proliferated in C3. Subclass C4 expressed high Th2 cells and the lowest levels of neutrophils, endothelial cells and fibroblasts. The heterogeneity of neutrophils in C1 and C4 could possibly be associated with the cancer progression of the subclasses, which we will discuss in the *Discussion* section. In addition, we explored the level of infiltrating immune cells versus stromal cells, and a significant difference was found among subtypes (**Figures 3D, E**, Kruskal– Wallis, P<0.001). C3 had the highest stromal score and immune score. Furthermore, we investigated the expression levels of immune-related genes (**Figure 3C**). C1 has low expression of checkpoint inhibitors such as LAG3, HAVCR2 and PDCD1, but it also expresses high levels of cytotoxic-related GZMA, PRF1, and CD8A. C3 expresses the high checkpoint inhibitors HAVCR2 and PDCD1. C4 is immune stimulatory and cytotoxically active, since CXCL9, CXCL10, IFNG, GZMA, GZMB and PRF1 were highly expressed. Finally, the landscape between the metabolic pathways and immune cells was visualized with a heatmap (**Figure S2B**).

### Correlation of GC subtypes with clinical characteristics

**Figure 4:**
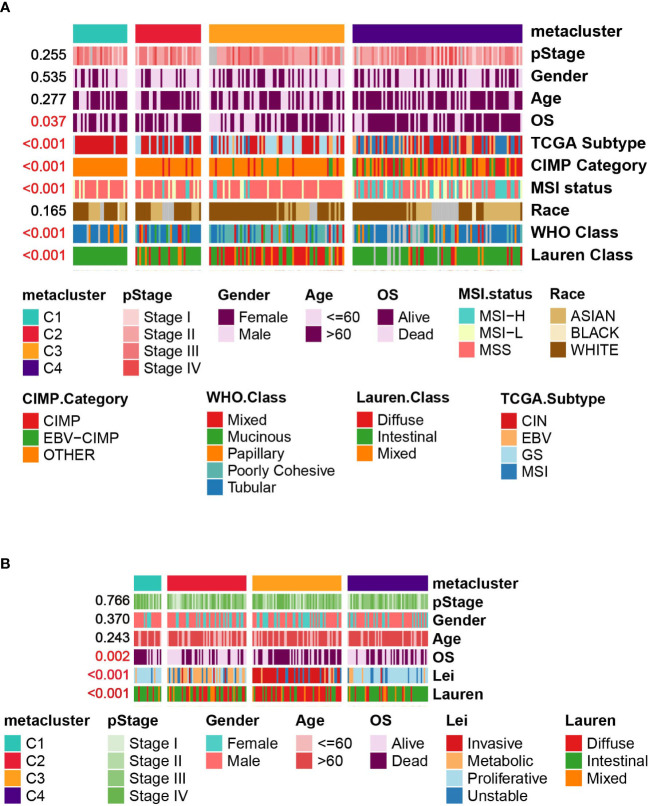
Correlation between GC subtypes and clinical characteristics in **(A)** TCGA cohort. **(B)** GSE15459 cohort.

We then investigated the associations between our metabolism-associated classification and other classification systems of GC. The comparison was performed separately for TCGA and GSE15459 cohorts (**Figures 4A, B** and **Tables S3**, **S4**). Significant correlations were found between our classification and TCGA subtype (Chi-square, P < 0.001), Lauren subtype (Chi-square, P < 0.001), and WHO subtype (Chi-square, P < 0.001). On the other hand, pStage, age, and sex were not correlated with subtype in either of the datasets. Notably, in the GSE15459 set, C2 and C3 highly mirrored the metabolic and invasive type by Lei’s classification. Such overlap is intriguing because the transcriptomic dataset and the clustering method were different. This finding suggested that metabolic transcripts could be the most informative in classifying gastric tumors.

### Correlation of GC subtypes with copy number variation, somatic mutation, tumor mutation burden and neoantigen

**Figure 5:**
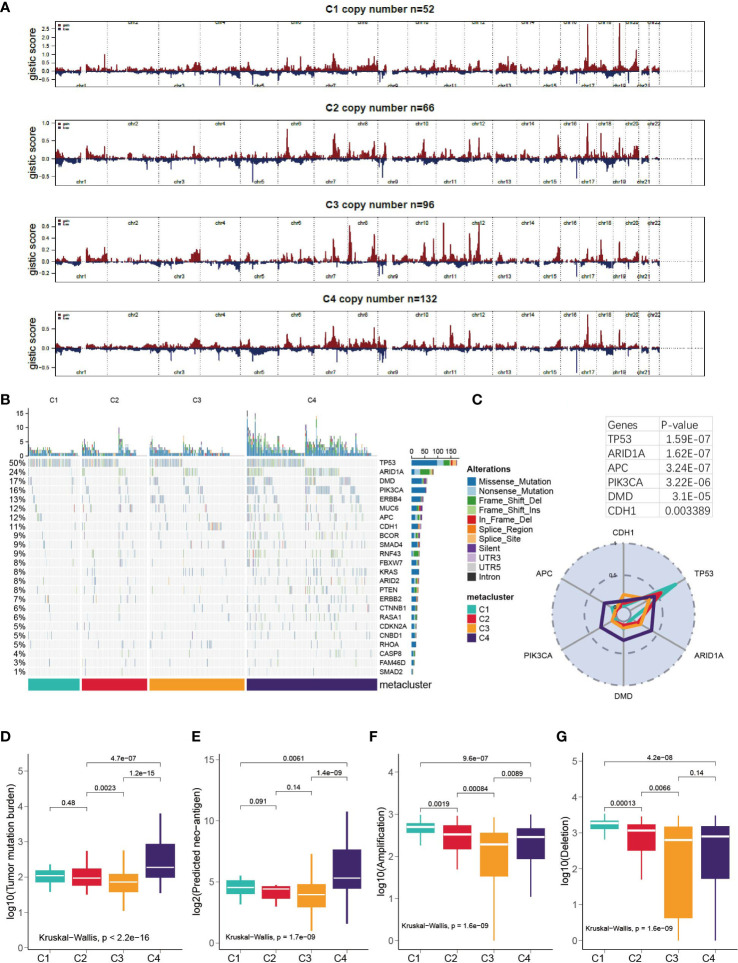
Correlation of GC subtypes with copy number variation, somatic mutation, tumor mutation burden and neo-antigen. **(A)** Visualization of the copy number variation, GISTIC plot for C1-C4. **(B)** oncoPrint for C1- C4. **(C)** Mutation rate of top mutated cancer driver gene in each subtype. **(D-G)** Correlation between subclasses and TMB, neoantigen, deletion and amplification.

First, we examined the landscape of the copy number variation of C1-C4 (**Figure 5A**). Then, the top mutated cancer driver gene was analyzed, led by TP53, ARID1A and so forth (**Figure 5B** and **Table S5**). TP53 was found to be mutated in 83% of C1 patients versus 56% in C2, 35% in C3 and 45% in C4. However, in general, C4 was the most mutated subtype because it had the highest mutation rate in many other genes (**Figure 5C**) and showed significantly higher TMB and neoantigen levels than the other subtypes (Kruskal–Wallis, P < 0.001, **Figures 5D, E**). In terms of amplification and deletion, C1 was significantly higher, while C3 was the lowest (Kruskal–Wallis, P < 0.001, **Figures 5F, G**). We further explored how the expression of the cancer driver genes relates to the prognostics (**Figure S3**). It was discovered that DMD was overly expressed in C3, and the median-based low DMD group had better overall survival in the training set (log-rank, P = 0.03); however, the curve did not separate significantly in the testing set. Additionally, the expression of CDH1 in C3 was inhibited, which could be associated with a high mutation rate of CDH1 in C3 (**Figure S3**).

### GSEA and enrichment analysis of DEGs

**Figure 6:**
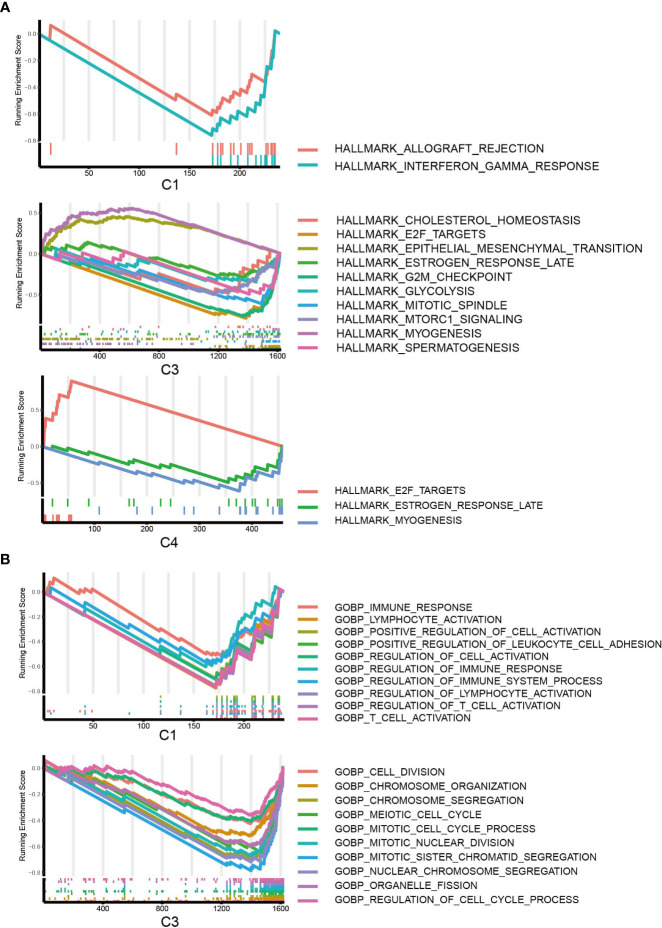
GSEA of DEGs. **(A)** GSEA of hallmark pathways for C1, C2 and C3; DEGs of C2 did not return with any pathways. **(B)** GSEA of GO pathways for C1, C3; C2 and C4 were not enriched for any GO pathways.

To further characterize the biological processes, we conducted GSEA based on the hallmark gene set (**Figure 6A**), Gene Ontology set (**Figure 6B**), and KEGG set, and we also performed GO and KEGG enrichment analyses (**Figures S4**, **S5A**). It should be highlighted that C1 was enriched for the downregulation of the interferon gamma response in the hallmark pathway and the KEGG pathways related to the immune response and antigen presentation. For C2, GSEA did not give any result, but C2 was enriched in several KEGG metabolism pathways (**Figure S5A**). The results suggested that C3 is enhanced for EMT and inhibited for glycolysis and cell division/cell cycle. C4 was enhanced for viral protein interaction (**Figure S5A**), which is associated with its higher EBV patient percentage.

### Correlation of HER2 and metabolism-related genes

**Figure 7:**
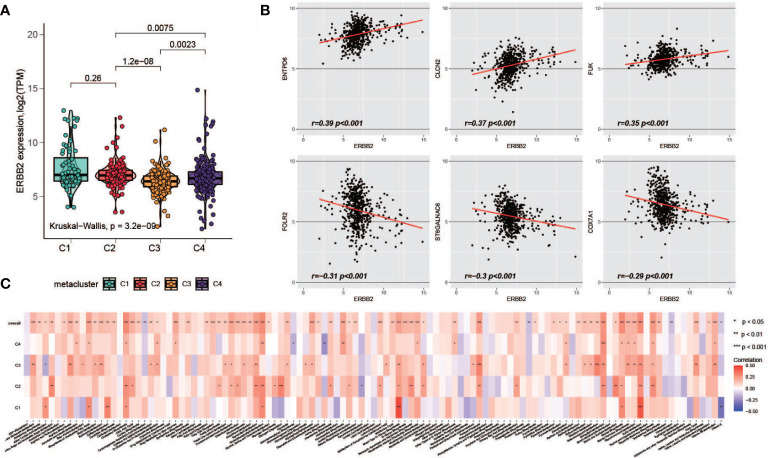
Association of HER2 and metabolism-related genes. **(A)** Comparison of HER2 expression level among C1-C4. **(B)** Top positively and negatively HER2-correlated metabolism-related prognostic genes **(C)** The correlation of HER2 with metabolism pathways in each metacluster.

HER2 overexpression is recognized as a frequent molecular abnormality drives gastric cancer, and it has been solidly correlated to poor disease outcomes (39, 40). Therefore, we investigated the HER2 expression with metabolism. Possibly owning to racial homogeneity, the expression of HER2 in cohort GSE84426 and GSE84433 has MAD less than 0.2, in order to be consistent with the data processing criteria, we performed the analyses of HER2 just with TCGA and GSE15459 datasets. First, we explored the expression of HER2 in the metaclusters. it was lower in C3 (**Figure 7A**). Next, we explored the correlation between the expression of HER2 and the enrichment of the metabolism pathways. We did not observe a pathway with correlation of opposite directions with HER2 among subgroups, however, the level of correlation is different across metaclusters (**Figure 7C**). Furthermore, we investigated the correlation between HER2 and 460 prognostic relevant metabolism-related genes. Top 3 positively and negatively correlated genes were plotted (**Figure 7B**) and the full results were provided in **Table S6**.

### Immunotherapy response prediction and drug sensitivity analysis

**Figure 8:**
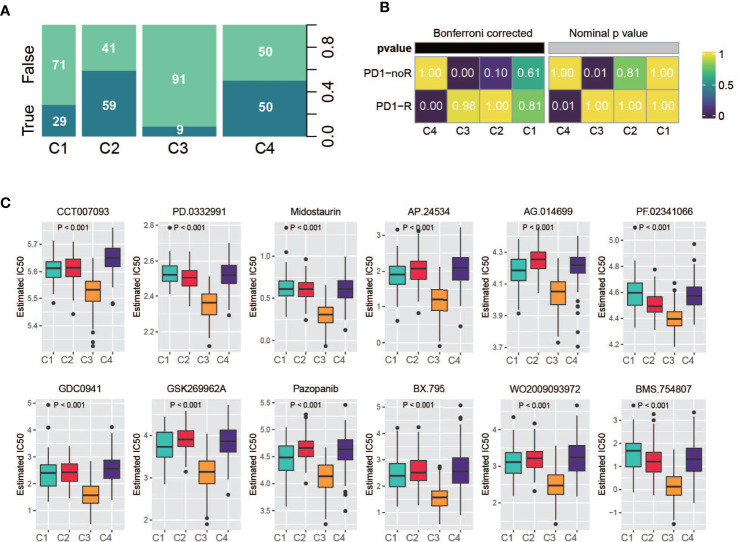
Drug sensitivity analysis. **(A)** Summarized result of TIDE analysis; the width of a cluster corresponds to the number of patients in that subtype; number represent the percentage of predicted responders/non-responders for each subtype. **(B)** SubMap analysis based on immunotherapy treated metastatic gastric cancer cohort, C4 shows high sensitivity. **(C)** Drug sensitivity based on Genomics of Drug Sensitivity in Cancer database, the top 12 drugs with the most significant p value were illustrated.

Recently, the FDA approved two PD-1 inhibitors (nivolumab and pembrolizumab) in combination with certain types of chemotherapy for the treatment of patients with metastatic gastric cancer/metastatic HER2-positive gastric cancer. Hence, we used multiple methods to investigate whether specific subtypes have prognostic value in predicting the response to immunotherapy. First, we utilized the TIDE online tool to predict patient response for each of the subtypes (33, 34). The classification was correlated with the predicted response to immunotherapy (**Figure 8A**, Fisher’s test, P < 0.001); a considerable portion of patients from C2 and C4 were predicted to have a response, while very few patients from C1 and C4 were identified as responders. Second, based on an anti-PD-1 immunotherapy-treated metastatic gastric cancer cohort (35), we investigated the response of the patients of a subtype to PD-1 treatment. As a result, C4 manifested a high likelihood of responding to PD-1 therapy, whereas the other subtypes did not (**Figure 8B**). In addition, we analyzed the sensitivity of subtypes of drugs in the Genomics of Drug Sensitivity in Cancer (GDSC) database. Among them, 270 drugs exhibited significantly different responses between the subtypes, and the top 12 drugs according to the p value (**Table S7**), indicating different responses, were plotted. C3 had the highest sensitivity of these drugs (**Figure 8C**).

### Subtype classifier and performance validation

**Figure 9:**
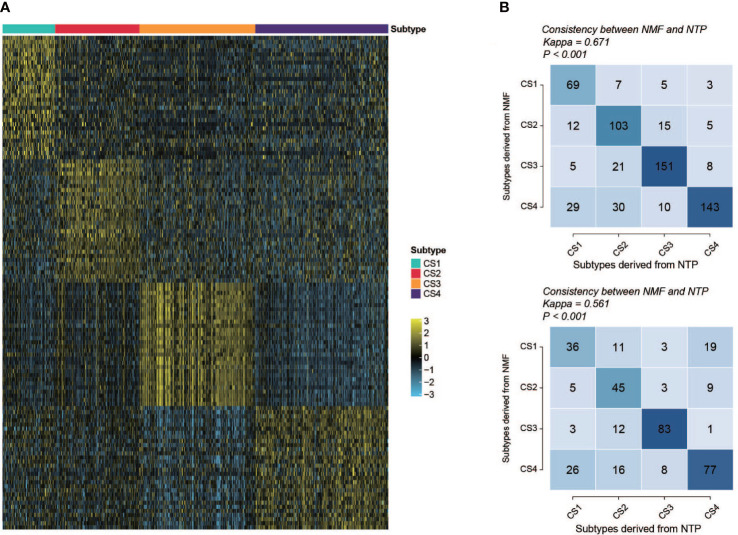
Performance and validation of the classifier. **(A)** Heatmap of the expression of the subclass classifier, 30 top up-regulated genes per subtype were illustrated. **(B)** Performance of the classifier (top, training set; bottom, validation set). Vertical axis shows the classification resulted from NMF; horizontal axis shows the classification predicted by NTP.

To create a classifier for clinical usage, it is required to choose top informative subclass-associated signature genes. After comprehensive consideration of accuracy and clinical application potential, a 120-gene classifier was generated and visualized (**Figure 9A**) based on the top 30 genes with the largest log2FC value (nominal P value < 0.05 and adjusted P value < 0.05) in each subclass (**Table S8**). Then, the performance of the classifier was examined with NTP in the training set and testing set separately. The prediction exhibited good overall concordance in the training set (kappa = 0.671, P < 0.001, **Figure 9B**, top) and moderate agreement in the testing set (kappa = 0.561, P < 0.001, **Figure 9B**, bottom). However, we also noticed that the prediction concordance for C1 and C4 was worse than that for C2 and C3, which we will discuss in the *Discussion* section.

### Development and validation of a metabolism-related prognostic signature

**Figure 10:**
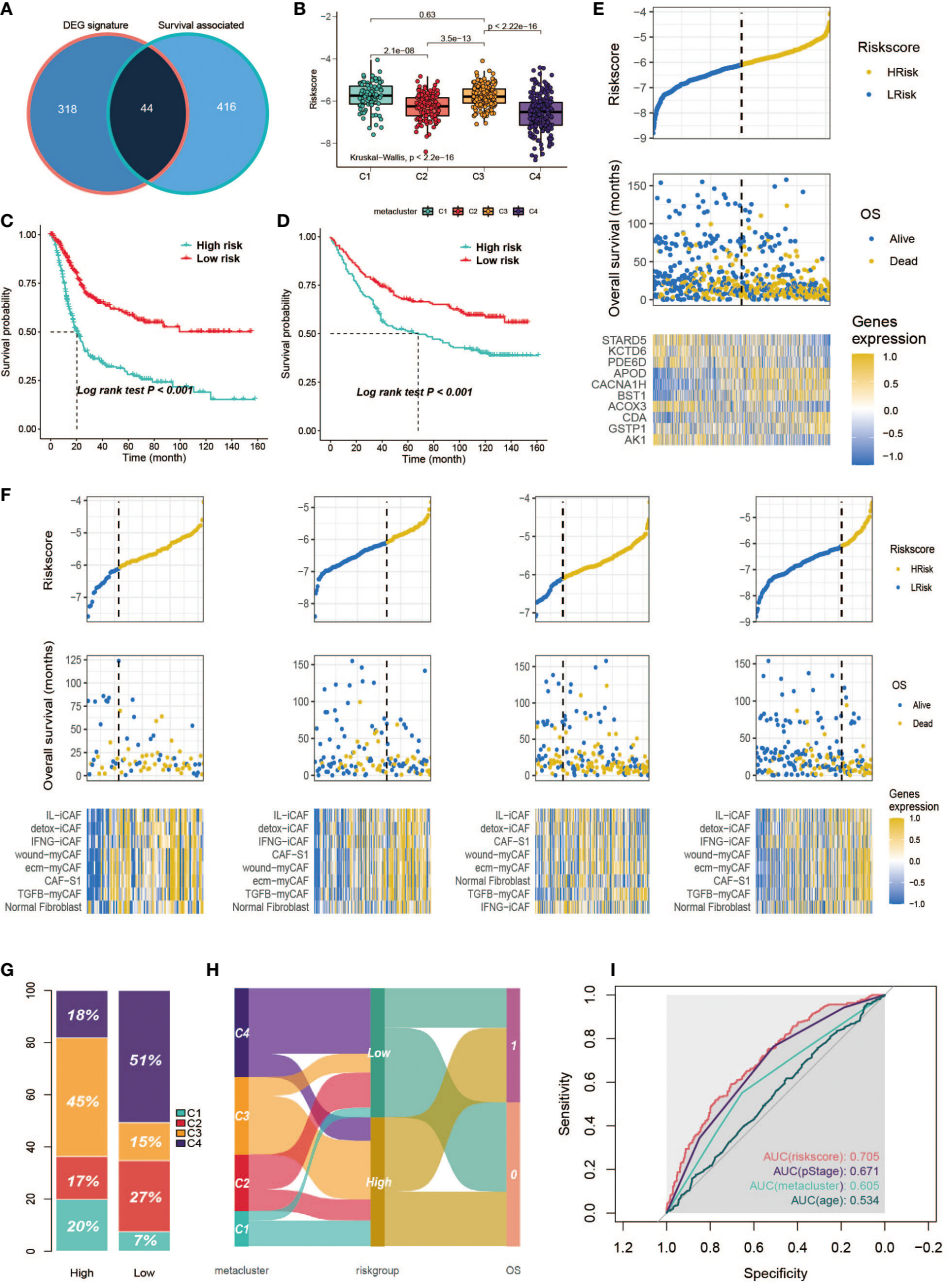
Development of a metabolism-related prognostic signature. **(A)** Venn diagram of DEGs and prognostic-relevant metabolism-associated genes. **(B)** Correlation between risk score and subclasses. **(C, D)** Kaplan-Meier plot of the patients in training set and GSE84433 data set. **(E)** Correlation between signature risk score and patients’ survival and risk signatures’ expression. Upper panel shows the distribution of the risk score; middle panel shows the patients’ survival status and time; bottom shows the heatmap of the risk signatures’ expression profiles. The black dotted line represents the median risk score cutoff dividing patients into low-risk and high-risk groups. **(F)** Correlation between signature risk score and patients’ survival and risk signatures’ expression in each subclass. Bottom panel heatmap of the fibroblasts’ expression profiles. **(G)** Distribution of the subclasses in high/low risk score groups. **(H)** ROC analysis of the risk score, pStage, age and metacluster. **(I)** Sankey plot of the relationship between subclasses and the risk group, and the relationship between risk group and OS.

Differential expression analysis followed by the Broruta process identified a total of 362 DEG signatures (**Table S9**), which were combined with the previously defined prognostic-relevant metabolism genes (**Figure 10A**) to fit the lasso-Cox model (**Figure S6A**). A 10-gene prognostic model was constructed (**Table S10**). A significant difference in risk score was observed among the subclasses (**Figure 10B**, Kruskal–Wallis, P < 0.001). As we would expect according to the survival curves of the subclasses, C1/C3 had a higher risk score than C4/C2. Then, patients were classified into high- and low-risk groups based on the median. The high-risk group exhibited a significantly poorer prognosis in both the training set (**Figure 10C**, log-rank, P < 0.001) and the testing set (**Figure 10D**, log-rank, P < 0.001). The risk score was plotted against the OS and the expression of gene signatures (**Figure 10E**). APOD, CACNA1H, BST1, CDA and GSTP1 are concentrated toward the high-risk end. APOD, CACNA1H, BST1 and CDA each independently correlated with OS (**Figure S6B**), suggesting the potential prognostic value of these genes. The expression of the prognostic genes in metaclusters was explored, APOD and CACNA1H were highly expressed by C3 (**Figure S6C**). We observed an association between the expression of fibroblasts and poor survival in C3 in a previous analysis. We are interested in determining whether it is a C3-specific feature or is global across all subclasses, and it was revealed that a negative association between fibroblasts and survival or risk score also existed in C1/C2/C4 (**Figure 10F**). In the high-risk group, 45% of the patients were from C3, and C4 contributed over 50% of the low-risk patients (**Figures 10G**, **H**). To further explore the prognostic value of the gene signatures, we performed ROC analysis to compare AUCs with age, pStage and metabolism-associated cluster (metacluster). The AUC of the risk score was 70.5%, which was higher than that of pStage (67.1%), metacluster (60.5%) or age (53.4%) (**Figure 10I**).

## Discussion

Our results showed that GC could be classified into four distinct metabolism-relevant subtypes, and the reproducibility of this subtyping was validated in the testing set. Each subtype was associated with different clinical characteristics, immune cell fractions and gene mutation alterations. First, the results showed that C1 displayed a low level of immune infiltration, as indicated by the low immune score and low expression of lymphocytes, e.g., T cells, B cells, NK cells, and myeloid dendritic cells. Downregulation of the interferon gamma response and immune response was discovered *via* GSEA. Therefore, we define C1 as an immune-suppressed subtype. Next, C2 was characterized by multiple metabolism KEGG pathways (**Figure S4A**), which is consistent with Lei et al.’s observation; thus, we continued to term it metabolic type. For C3, endothelial/epithelial-mesenchymal transition is strongly evident by high levels of endothelial cells, fibroblasts, low CDH1 expression, and the EMT hallmark pathway, so we term it the mesenchymal type or immune exhausted type from an immune infiltration point of view. Finally, C4 is characterized by high levels of neoantigen, TMB, cancer driver gene mutation and MSI; therefore, we term it the hypermutated type.

There have been numerous studies about gastric cancer classification, and our study pursued this topic from a metabolism angle. The results manifested a clear link to Lei’s research. Superficially, C1 and C4 jointly correspond to Lei’s proliferative subtype, indicating that in broad transcriptomic terms, C1 and C4 share molecular similarities. This may help to explain why the classifier is less accurate in distinguishing these two subtypes. For example, 26 out of 127 C4 patients were misclassified as C1 in the testing set, and 19 out of 69 patients in C1 were mislocated as C4 in the testing set. However, we argue that C1 and C4 are heterogeneous. First, subtype C1 is highly enriched in the WNT signaling pathway but poorly enriched in the NR2F pathway, which is the opposite of the C4 subtype, implying they have different oncogenesis mechanisms. In addition, C1 shows signs of a cold tumor and is unlikely to respond to immunotherapy, whereas C4 is immune active. It could be hypothesized that the difference in metabolism signatures observed between C1 and C4 reflects the discrepant behavior of infiltrated immune cells, which could be further studied in the future. Moreover, we hypothesize that the immune suppression in C1 is driven by the abundant infiltrated neutrophils. This phenomenon has been reported by several studies by stimulating the JAK-signal transducer and activator of STAT3 signaling pathways. Tumor-derived granulocyte-macrophage colony-stimulating factor efficiently activated neutrophils and triggered PD-L1 expression on neutrophils, which further suppressed normal T cell immunity and was associated with disease progression in GC (37, 41).

Subtype C4 has exhibited highly mutative nature out of the classification, as it expresses significantly high TMB and neoantigens and consists of a higher proportion of MSI-high patients. Somatic mutations are capable of encoding alien immunogenic antigens or neoantigens; thus, cancers with a high number of somatic mutations as a result of mismatch repair deficiencies might be vulnerable to immune checkpoint inhibition. Several clinical trials have demonstrated that MSI-high status is associated with prolonged progression-free survival (42–44), including GC (45, 46). However, according to TCGA classification, MSI-H only consists of 22% of gastric cancers (14), and a more common biomarker for ICI treatment in GC is needed. Tumor mutation burden has been shown to have a predictive effect on prognosis in multiple cancer types for immunotherapy (47–49), and a recent clinical trial of toripalimab confirmed the association between TMB-high and OS in GC (50). Except for these genomic alteration characteristics, C4 contains the majority of EBV-infected patients; in a recent study, six out of six EBV-positive patients treated with pembrolizumab exhibited remarkable responses (35). Taken together, these points suggest that C4 is inclusive of multiple confirmed predictive factors, e.g., TMB, MSI, neoantigen and EBV. Thus, we suggest this classification can be used as a predictive tool for immunotherapy.

Our study showed that a high level of fibroblasts is associated with poor prognostics between subclasses and within subclasses. Hence, we hypothesize that cancer-associated fibroblasts (CAFs) play a crucial role in modulating the TME in GC. First, it has been reported that in GC, CAFs produce fibroblast activation protein alpha (FAP), which promotes cancer progression *via* EMT through the WNT/β-catenin signaling pathway (51). This mechanism is confirmed by our result that C3 exhibited an enhanced EMT process and high enrichment of the WNT pathway. Second, echoed by other studies, high expression of CAFs is linked to an immune suppressive tumor microenvironment, which can be further linked to poor survival outcomes (52). This is consistent with our observation in C3, as well as in the other three subtypes; higher fibroblast levels worsened OS. In addition, our results suggest that the TGFβ-dependent signaling pathway is involved in immunosuppression of the C3 subtype, as evidenced by high enrichment in the TGF-pathway. TGF changes the function of CD8+ T cells by suppressing the production of critical genes involved in their cytotoxic action. By secreting TGF and IL6, -SMA+ FAP+ CAFs in head and neck cancer suppress the growth of CD8+ T cells and increase the recruitment of CD4+ CD25+ T cells (53). Furthermore, it was recently discovered that CAFs from breast, ovarian, lung, pancreas and colon cancer express PD-L1 and/or PD-L250 (54–57) particularly in the FAP-high CAF subset (54, 55). These ligands bind to the PD-1 receptor expressed by T cells and inhibit T cell activity (52, 54, 57, 58). Therefore, anti-CAF therapy can be considered as an addition to current treatment for GC, which requires more research.

In summary, we identified the prognostic value of metabolic-gene-associated GC subclasses and proposed a 120-gene classifier for immune-suppressed (C1), metabolic (C2), mesenchymal/immune exhausted (C3) and hypermutated (C4) subtypes. The metabolic and hypermutated subtypes have better overall survival, and C4 is likely to be sensitive to anti-PD-1 immunotherapy. The metabolism-associated classification can be used to stratify patients and identify those who will benefit more from immunotherapy. In addition, a risk score based on a 10-gene signature was developed for GC patient survival time prediction. We further identified the immune landscape, signaling pathways and clinical characteristics for each subclass, deepened our understanding of the heterogeneity of GC, and provided information for the development of new strategies for GC treatment.

## Data availability statement

Publicly available datasets were analyzed in this study. This data can be found here: TCGA (https://portal.gdc.cancer.gov/) with cancer type of STAD; GEO data repository (GSE15459, GSE84426 and GSE84433; https://www.ncbi.nlm.nih.gov/gds).

## Author contributions

YY, WY, and XR executed the data analysis and data interpretation; WY, LX, WC, XW, and MZ participated in the graphing and manuscript writing; XC, DC, GL, and YW collected, processed and cleaned the data; FY, XL, and LJ supervised the project and provided guidance. All authors contributed to the article and approved the submitted version.

## Funding

This work was supported by the National Key R&D Program of China (2019YFC1711000), National Natural Science Foundation of China (81973145), Key R&D Program of Jiangsu Province (Social Development) (BE2020694), and Active Components of Natural Medicines and Independent Research Projects of the State Key Laboratory in 2020 (SKLNMZZ202016).

## Acknowledgments

We greatly appreciate the patients and investigators who participated in the corresponding medical project for providing data.

## Conflict of interest

The authors declare that the research was conducted in the absence of any commercial or financial relationships that could be construed as a potential conflict of interest.

## Publisher’s note

All claims expressed in this article are solely those of the authors and do not necessarily represent those of their affiliated organizations, or those of the publisher, the editors and the reviewers. Any product that may be evaluated in this article, or claim that may be made by its manufacturer, is not guaranteed or endorsed by the publisher.
