# Supplementary material for: Metabolism-associated molecular classification of gastric adenocarcinoma
Source: Front Oncol. 2022 Nov 16;12:1024985. doi: 10.3389/fonc.2022.1024985 (PMC9709214; doi:10.3389/fonc.2022.1024985)
Supplement: Supplementary file 1 [file DataSheet_1.docx]

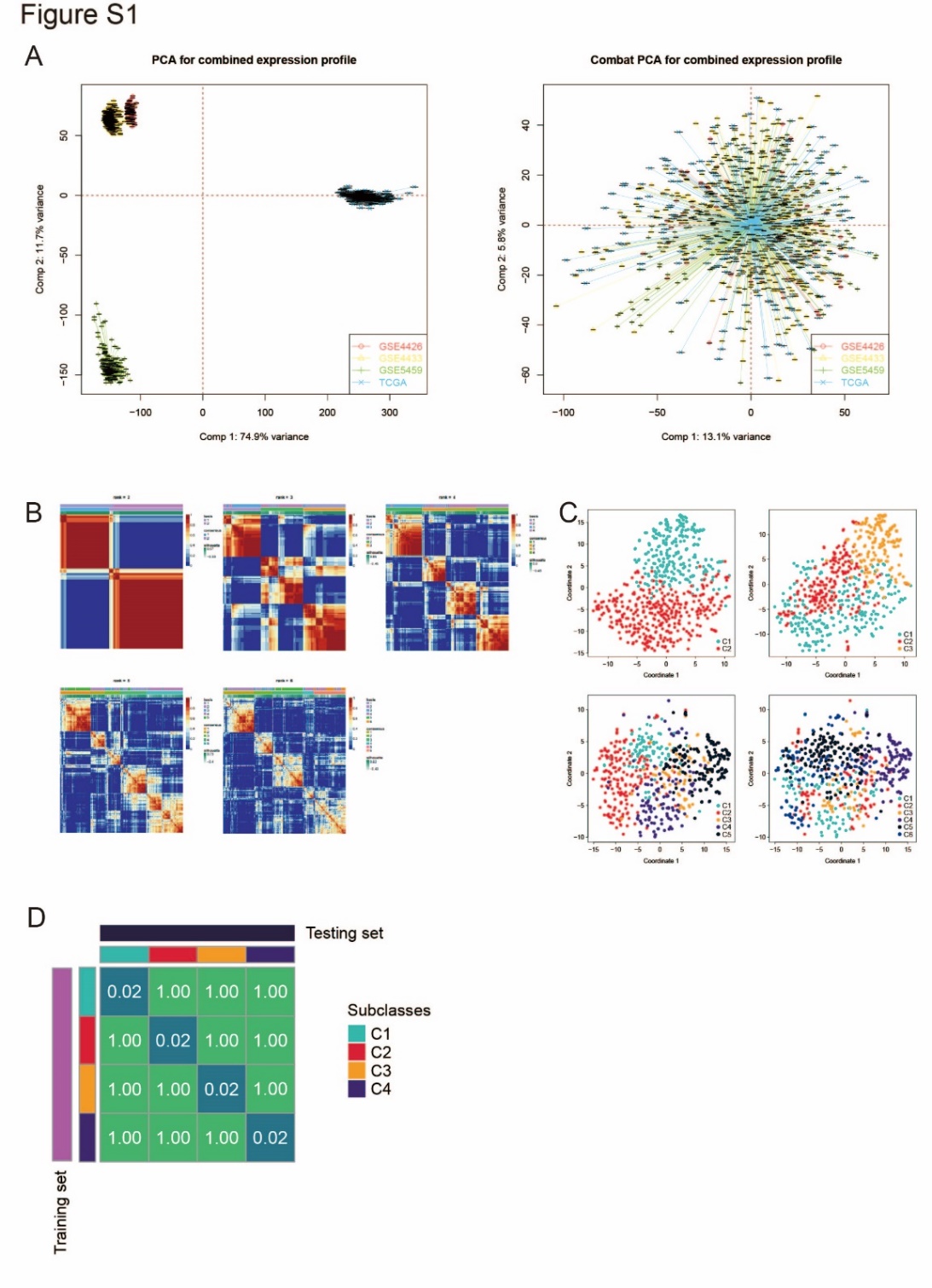
Supplementary Figures

Supplementary Figure 1. (A) Diagnostics of the batch effect correction with PCA. Left and right panel shows the PCA result before and after the batch effect correction, the samples were well mixed after batch effect removal. (B) The consensus matrix of the NMF clustering for k= 2 to 6. (C) t-SNE plot for cluster number 2, 3, 5 and 6. (D) The results of Submap analysis. The elements on the diagonals were significant, indicating pairwise similarity between the subclasses in the training set and validation set.


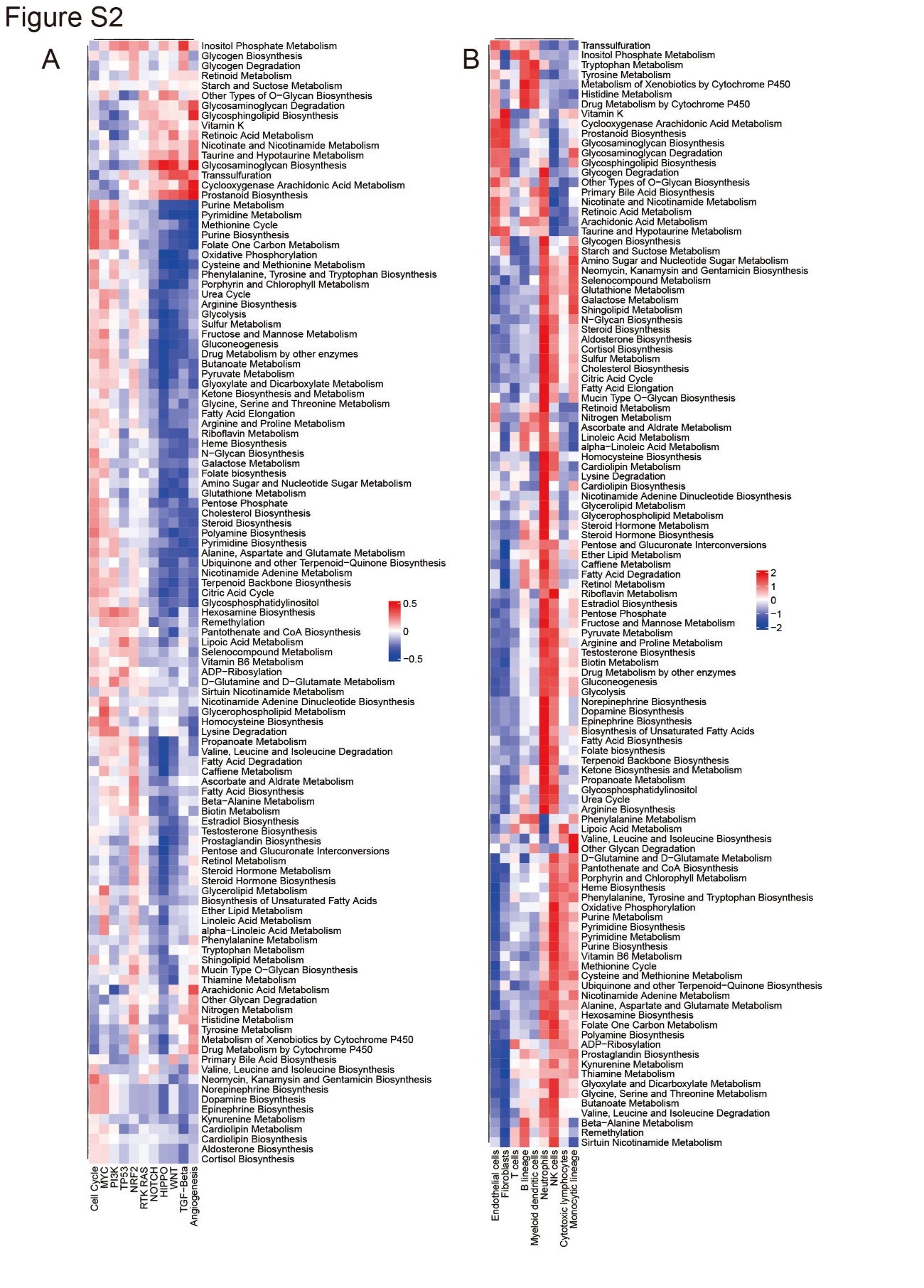


# Supplementary Figure 2. Heatmap of the correlation between metabolism pathways and (A) oncogenic pathways (B) immune cell abundances.


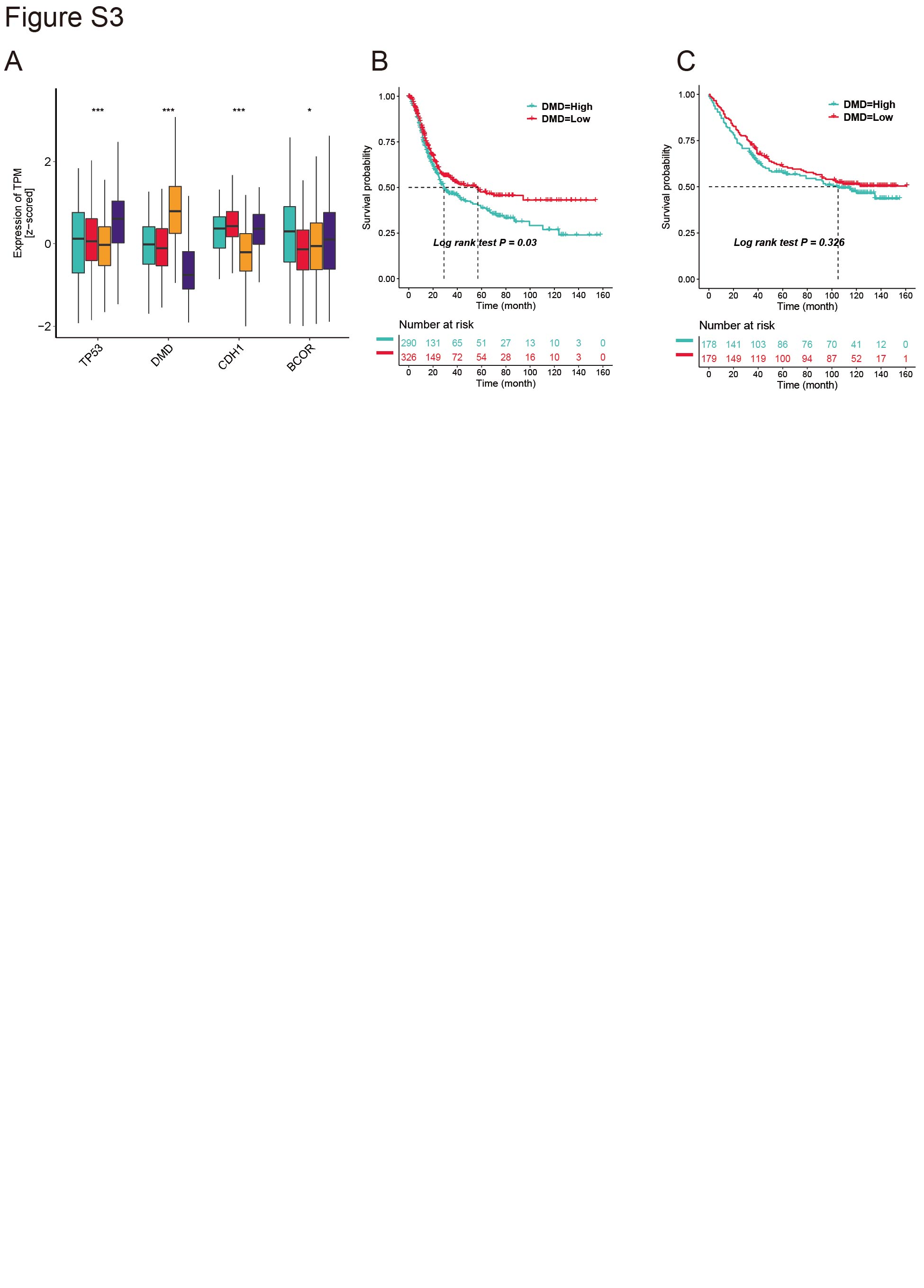
Supplementary Figure 3. (A) Boxplot of the cancer driver gene expression with respect to metacluster. (B) Kaplan-Meier plot of the patients in high/low DMD group in training set (left) and validation set (right), median was used as cutoff.


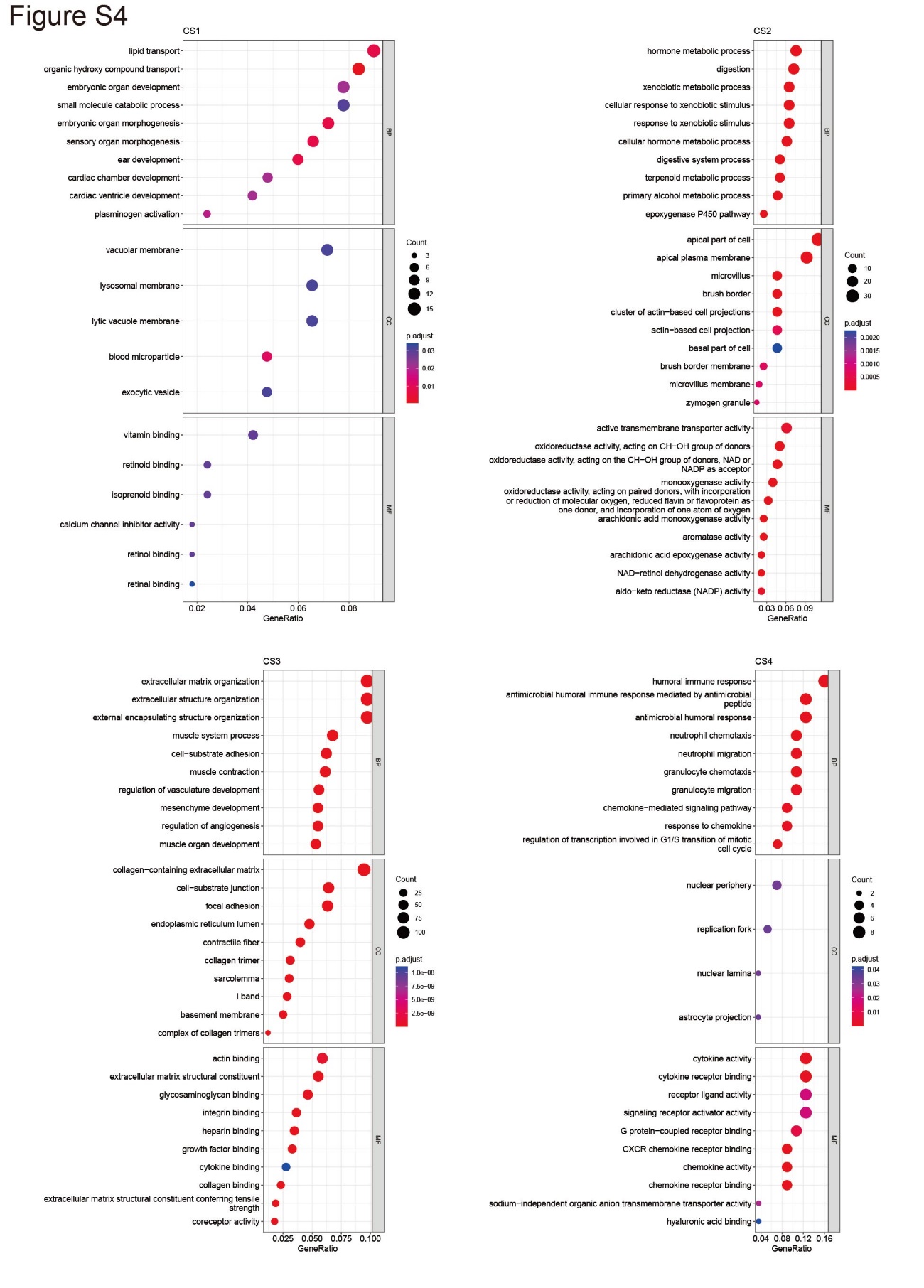


# Supplementary Figure 4. GO enrichment of DEGs


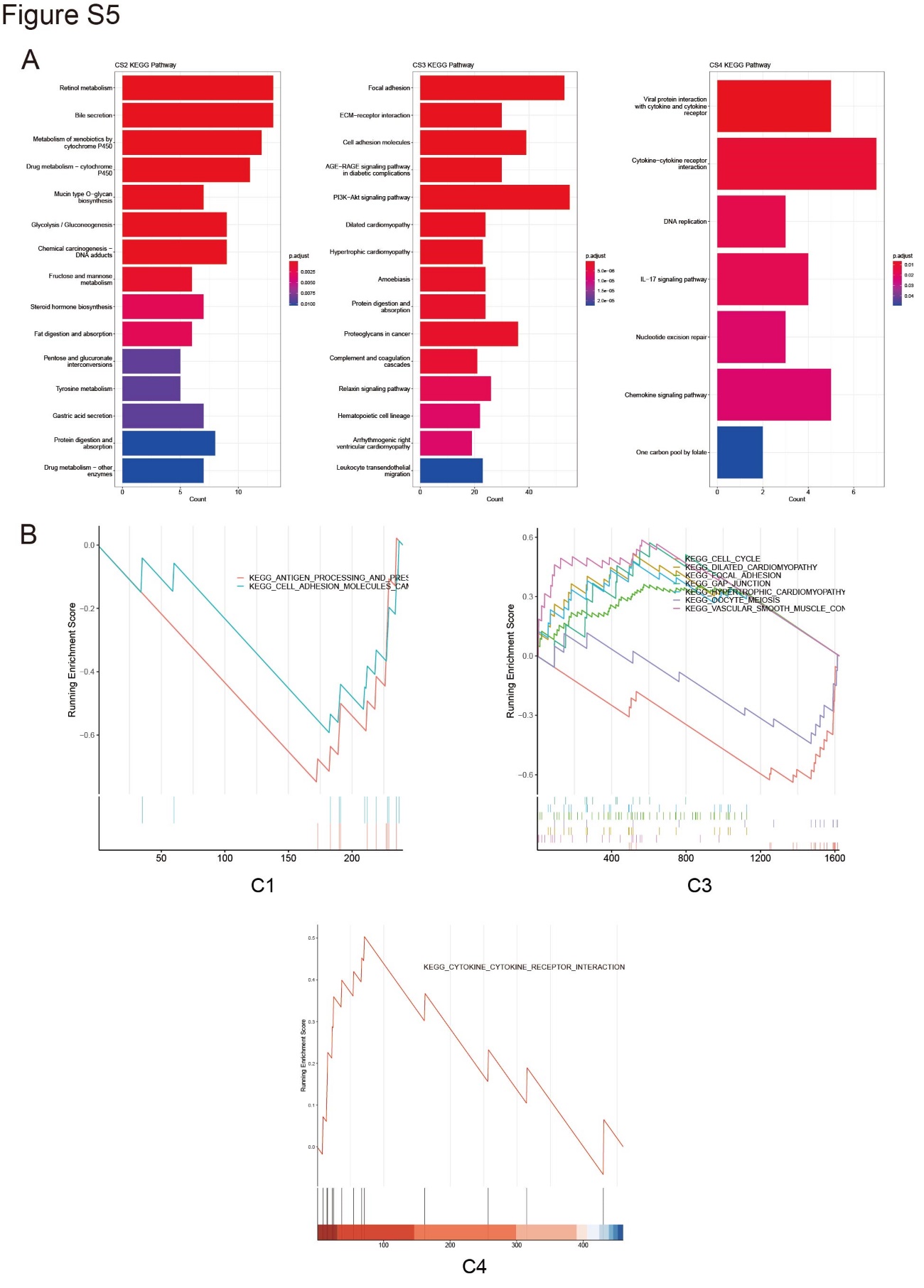


# **Supplementary Figure 5.** KEGG enrichment (A) and GSEA of DEGs (B)


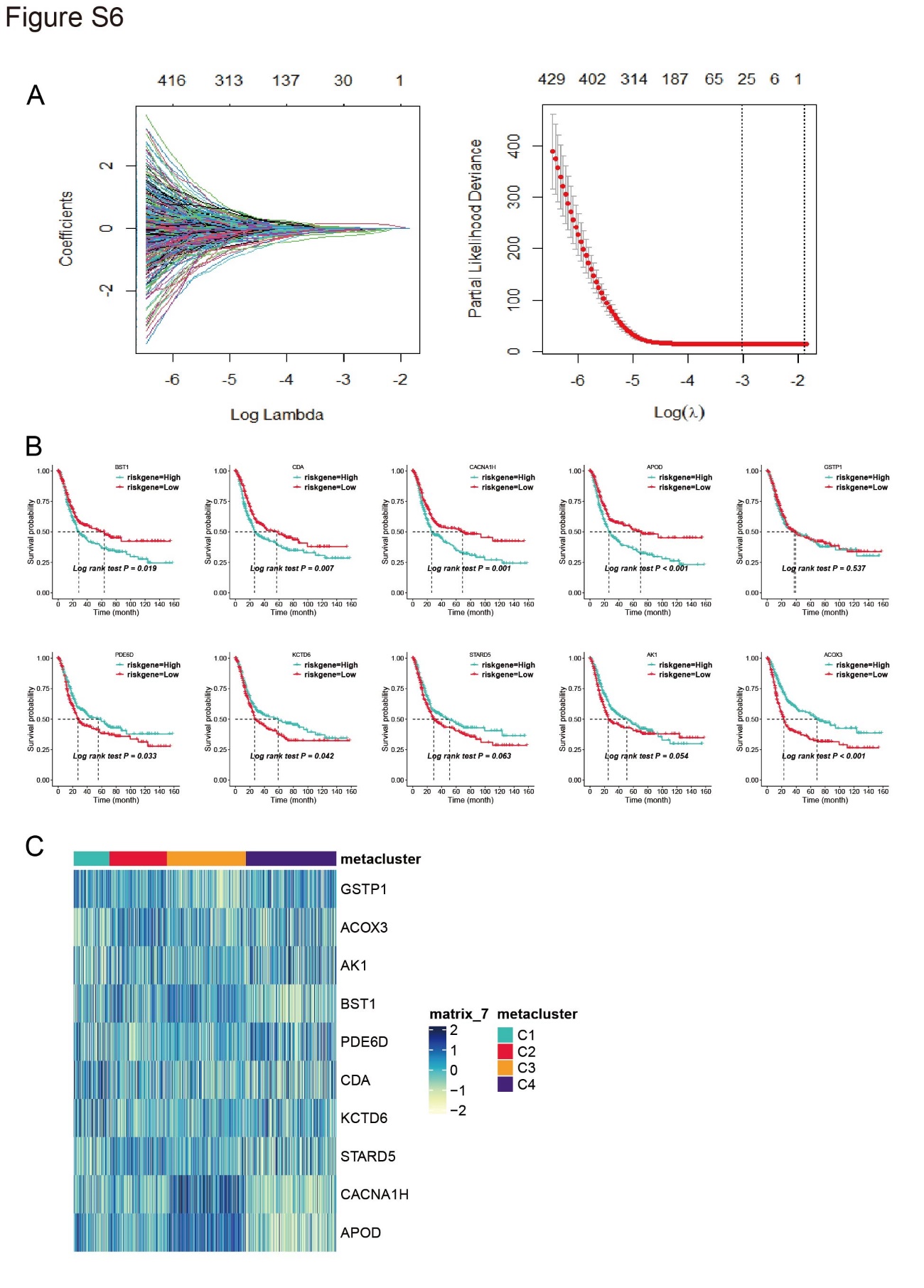


Supplementary Figure 6. (A) Model diagnostics of the lasso cox model. Association between the number of coefficients and the value of lambda (left); 10-fold cross validation was used (right). (B) The prognostic value of individual prognostic gene. (C) Expression heatmap of the prognostic signatures.
